# Royal Hospital for Incurables

**Published:** 1893-12-02

**Authors:** 


### ROYAL HOSPITAL FOR INCURABLES.

The annual meeting in connection with this institution—situate at West Hill, Putney Heath—was held on Friday at the City Terminus Hotel, Cannon Street, Mr. William D. Freshfield, vice-president, in the chair. The Secretary, Mr. Frederic Andrew, read the report, in which great regret was expressed at the death of Mr. William M. Seaman and Mr. J. Derby Allcroft. Lord Aberdeen had retired from the office of president on his appointment as Governor-General of Canada, but in resigning the appointment his lordship stated that he would continue to take a deep interest in the welfare of the hospital. The finances compared unfavourably with those of the previous year, the income falling short by no less than £5,000, and the balance at the bankers at the close of the financial year was totally inadequate to pay current expenses. The entire receipts for the year ended September 30th were £29,111, while the disbursements amounted to £34,615. With respect to the criticisms on the institution made in 1891 by the Select Committee of the House of Lords on the Management of Metropolitan Hospitals, a committee of inquiry had examined into the charges and taken evidence upon them, the result being that the charges had been disproved. The report of the committee of inquiry was unanimous on the part of those who attended, with the exception of Lord Aberdeen and Lord Arran, who did not agree with some of the conclusions of their colleagues. The report of the committee was afterwards read, which set out the five specific reforms recommended by the Lords Committee—that a resident medical officer should be appointed, that a ladies' committee should be appointed, that all nurses should be hospital trained, that the contracts for food and stores of all kinds should be by open tender, and that the general supervision by the committee of governors should be greatly increased. The committee of inquiry set out reasons why in their opinion no alterations were necessary in any of these directions. Lord Aberdeen, however, thought "that an opportunity should be taken, as soon as such occurs," for appointing a resident medical officer, and that, "however valuable and beneficial the visits of ladies as at present to the inmates of the institution may be, it would be desirable that a certain number of ladies should be asked to join the board or committee of management." Lord Arran was also of opinion that a resident medical officer should be appointed. This proposal was objected to by Mr. Freshfield, on the ground that the man they would appoint would be a young medical man eager to put all things to rights. He would leave them in about three years, but not until he had given them a great deal of trouble. Lord Arran went on to say that there was no reason to doubt the efficiency of the present staff of female nurses, but he suggested that the staff should be recruited from as large a number of hospitals as possible. The adoption of the report and financial statement was proposed by the Chairman, who spoke of the need in which the hospital stood of additional pecuniary aid. They had suffered from the bad times and the report of the Lords Committee. The motion was seconded by Mr. Kimber, M.P., who asked those present to dismiss from their minds the idea that there was any justice whatever in the criticisms of the Lords Committee. A poll was afterwards taken, when 30 candidates were elected to the benefits of the institution out of a list of 149.

